# Novel adhesive mineral-organic bone cements based on phosphoserine and magnesium phosphates or oxides

**DOI:** 10.1007/s10856-023-06714-6

**Published:** 2023-03-24

**Authors:** Tobias Renner, Paul Otto, Alexander C. Kübler, Stefanie Hölscher-Doht, Uwe Gbureck

**Affiliations:** 1 Department for Functional Materials in Medicine and Dentistry, University Hospital Würzburg, Pleicherwall 2, 97070 Würzburg, Germany; 2 Department of Oral & Maxillofacial Plastic Surgery, University Hospital Würzburg, Pleicherwall 2, 97070 Würzburg, Germany; 3 Department of Trauma, Hand, Plastic and Reconstructive Surgery, University Hospital of Würzburg, Oberdürrbacherstraße 6, 97080 Würzburg, Germany

## Abstract

Present surgical situations require a bone adhesive which has not yet been developed for use in clinical applications. Recently, phosphoserine modified cements (PMC) based on mixtures of o-phosphoserine (OPLS) and calcium phosphates, such as tetracalcium phosphate (TTCP) or α-tricalcium phosphate (α-TCP) as well as chelate setting magnesium phosphate cements have gained increasing popularity for their use as mineral bone adhesives. Here, we investigated new mineral-organic bone cements based on phosphoserine and magnesium phosphates or oxides, which possess excellent adhesive properties. These were analyzed by X-ray diffraction, Fourier infrared spectroscopy and electron microscopy and subjected to mechanical tests to determine the bond strength to bone after ageing at physiological conditions. The novel biomineral adhesives demonstrate excellent bond strength to bone with approximately 6.6–7.3 MPa under shear load. The adhesives are also promising due to their cohesive failure pattern and ductile character. In this context, the new adhesive cements are superior to currently prevailing bone adhesives. Future efforts on bone adhesives made from phosphoserine and Mg^2+^ appear to be very worthwhile.

**Graphical Abstract:**

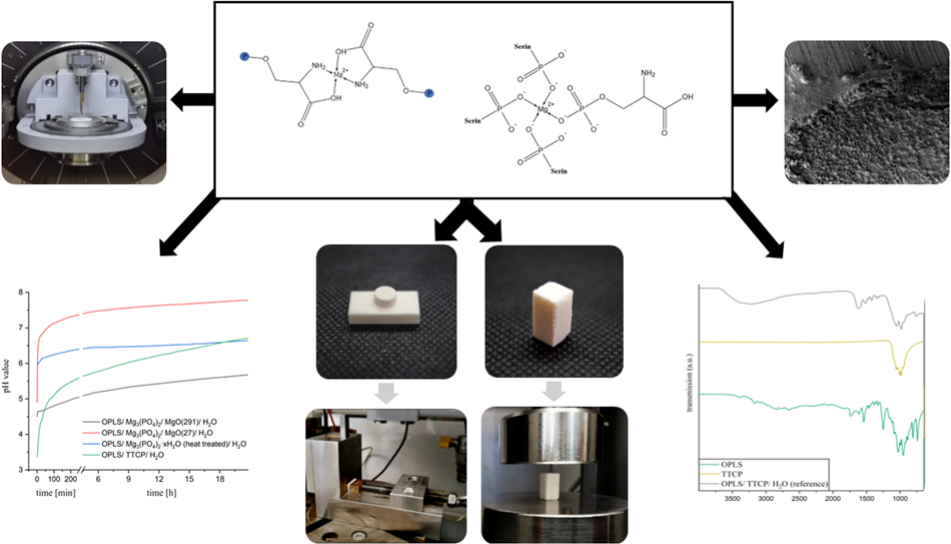

## Introduction

The fixation of small fragments, such as in comminuted fractures, currently presents surgeons with a major challenge. Some fracture configurations cannot be repaired at all, or only inadequately with currently used methods such as plate and screw osteosynthesis [1]. Therefore, the development of an absorbable bone adhesive, especially for fixing small unloaded fragments has long been desired [2–4] but not established in routine practise [5]. The reason for this is that the development of a bone adhesive is far from trivial. Necessary requirements are cyto- and biocompatibility, biodegradability and the replacement of the adhesive by new bone tissue [5]. These challenges, as well as the difficult curing conditions, challenge researchers. While polymeric adhesives based on polymethylmethacrylate (PMMA) bone cements or various cyanoacrylates show sufficient adhesive strengths of up to 0.35–1.9 MPa (PMMA) [6–8] and 0.16–12.1 MPa (cyanoacrylates) [8–12], they are not biodegradable and therefore present barriers with respect to bony remodelling processes. PMMA can also lead to bone necrosis via monomer release and heat generation during polymerization [13]. Cyanoacrylates specifically lead to cytotoxic and other adverse biological processes [14–16]. In contrast, biocompatible bone adhesives, such as aluminum-free glass ionomer cements [17], fibrin glue [8, 12] or gelatine-resorcine-formaline (GRF) based adhesives [12], have significantly lower bond strengths to bone and are obviously inferior to the formulations studied.

Various mineral bone adhesives have been investigated, which are commonly based on a chelation reaction between a calcium or magnesium phosphate and an organic component bearing various chelating moieties such as phosphate, carboxylate or amino groups. Brückner et al. [18] investigated a magnesium phosphate cement containing farringtonite (Mg_3_(PO_4_)_2_), MgO, and phytic acid as chelating agent and achieved an adhesive strength of 0.81 ± 0.12 MPa on cortical bone substrates after one week of storage in PBS buffer [18]. Other approaches are based on the reaction of various calcium phosphates such as α-tricalcium phosphate (α-TCP) or tetracalcium phosphate (TTCP) with ortho-phospho-L-serine (OPLS), named as Tetranite® [19] and OsStic™ [20]. Here, adhesive strengths of up to 2.5–4 MPa were recorded for a combination of phosphoserine and α-TCP when cured in an aqueous environment [21], while the reaction between TTCP and OPLS resulted in an adhesive strength of ~2 MPa to cortical bone substrates [22]. The use of a phosphorylated amino acid in these bone adhesives is not arbitrary. Phosphoserine is an important component of non-collagenous proteins of bone metabolism such as osteopontin or bone sialoprotein and mediates interactions with the hydroxyapatite crystals of the inorganic bone matrix [23–27]. Bone cements modified with phosphoserine showed increased osteoblast proliferation and differentiation, enhanced monocyte activation and, in animal studies, improved osteoconduction, as well as a generally increased bone remodelling rate [28]. Phosphoserine is also assumed to play a major role in organic adhesives produced by animals, such as the common blue mussel [29–31], the sandcastle worm [32–36] or the caddis fly [37].

The aim of this work was to investigate new mineral-organic bone cements based on phosphoserine and magnesium phosphates or magnesium oxides. These are thought to possess strong adhesive properties since Mg^2+^ in biocomplexes demonstrate a strong complexation to the phosphate group of phosphorylated amino acids which, depending on the pH, partly does not complex at all [38]. Suitable formulations were identified, which were analyzed by X-ray diffraction, Fourier infrared spectroscopy and electron microscopy and subjected to mechanical tests. A novel mechanical test method was used to determine the bond strength to bone. Different calcined magnesium oxides with varying reactivity were used in the determination of suitable adhesive cement compositions. In addition, various magnesium phosphates, such as tribasic magnesium phosphate hydrate or farringtonite, were used. The advantages and adhesive mechanical superiority over previously used bone adhesives were discussed and the special potential of this new system was elaborated.

## Materials and methods

For the preparation of farringtonite raw powder, magnesium hydrogen phosphate (Sigma-Aldrich/ Honeywell, Missouri, United States) was sieved to ≤125 µm (Retsch Technology GmbH, Haan, Germany) and the obtained powder was dry ground in an agate jar for one hour at 200 rpm. The ground powder was mixed with magnesium hydroxide (VWR Prolabo, Pennsylvania, United States) in a 2:1 molar ratio and the mixture was sintered at 1100 °C for five hours, followed by grinding and sieving it to ≤355 µm. Finally, the powder was dry ground again for one hour. The commercially available Mg_3_(PO_4_)_2_∙xH_2_O (Acros Organics, New Jersey, United States) raw powder was heat treated in a high-temperature furnace (Nabertherm GmbH, Lilienthal, Germany) at 400 °C for 6 h. TTCP powder was synthesized from a mixture of 12.1 mol CaHPO_4_ (Fluka Honeywell, New Jersey, United States) and 11.5 mol CaCO_3_ (Merck, Darmstadt, Germany) by sintering at 1500 °C for five hours. Finally, sieving to 125 μm was performed to the TTCP powder.

The following powders were used as reactants: crystalline farringtonite, temperature-treated TMP hydrate, and two magnesium oxides with different reactivity (Magnesia 291 and Magnesia 27, Magnesia GmbH, Lüneburg, Germany). These were combined with phosphoserine (Sigma-Adrich GmbH, Steinheim, Germany) and a reaction was induced by the addition of water. When producing the adhesive cements the dry mass was always mixed homogeneously in advance. All components were mixed rapidly on a glass plate with a spatula. In preceding phases of the experimental determination to identify suitable cement compositions, a defined quantity of OPLS was first methodically mixed in systematic series with different quantities of magnesium oxide and/ or magnesium phosphate raw powders, which in turn were then tested in different ratios to one another. The dry mixes were each combined with water at different PLRs. The presumed effects of individual parameters on the cement’s properties were recorded. Most of the compositions were unusable as bone adhesives. In most cases they did not have a suitable viscosity, cured too fast, too slowly or not at all, or apparently had no adhesive properties. When a paste with suitable characteristics occurred, it was immediately either filled into moulds or bone specimens were bonded with it. Suitable characteristics were: an uncomplicated homogeneous mixing to the cement paste, a processing time of 30 s—1 min, a dimensional stability of the cement mass after 2–3 min and a maximum setting time of 15 min (at 37 °C). In addition, the mixed paste should be low viscosity to transfer into a 1 ml injection syringe for precisely applying to the bone specimens.

In summary, three compositions of adhesive cements with suitable properties were investigated in this work: Composition (**1**): 1.38 mmol OPLS, 0.9 mmol farringtonite, 2.23 mmol MgO (291), PLR 3.87 g/mL; composition (**2**): 1.38 mmol OPLS 0.78 mmol farringtonite, 3.23 mmol MgO (27), PLR 3.93 g/mL; composition (**3**): 0.81 mmol OPLS, 1.52 mmol TMP hydrate (heat treated), PLR 2.56 g/mL. A reference composition (**4**) originates from the reference [39]. It contains 1.09 mmol (400 mg) TTCP, 0.81 mmol (150 mg) phosphoserine, and a PLR 4.23 g/ml and thus no magnesium phosphate or oxide. All the cement formulations used are listed in Table 1.

**Table 1:** Suitable compositions, which have been subjected to mechnical tests on bone, as well as to analyses of the setting behaviour and further analyses

Sample designation	Composition		PLR
(1)	OPLS	255 mg	3.87
	farringtonite	235 mg	
	MgO(291)	90 mg	
	H_2_O	150 μl	
(2)	OPLS	255 mg	3.93
	farringtonite	205 mg	
	MgO(27)	130 mg	
	H_2_O	150 μl	
(3)	OPLS	150 mg	2.56
	TMP∙H_2_O (heat treated)	400 mg	
	H_2_O	215 μl	
(4) [39] (reference)	OPLS	150 mg	4.23
	TTCP	400 mg	
	H_2_O	130 μl	

**Fig. 1:**
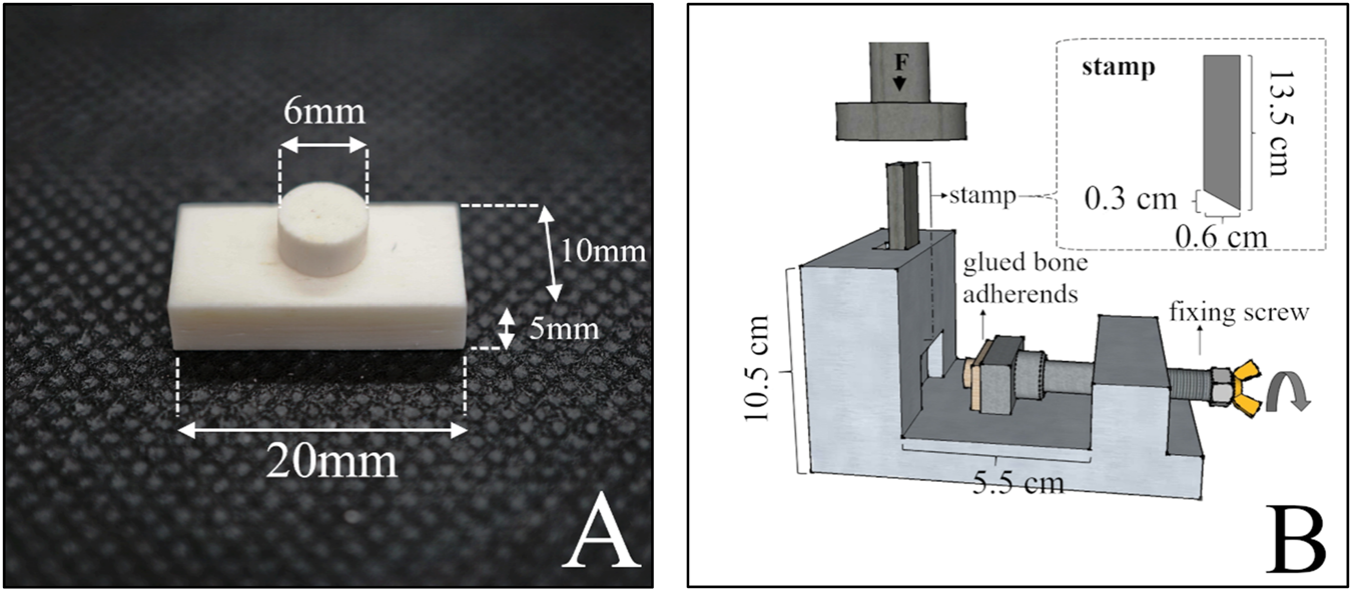
Bonded bone test specimens for testing the bond strength (**A**) and set-up of the shear test fixture (**B**)

The test specimens were prepared according to standard procedures from fresh bovine femurs with the diaphysis cut into coarse pieces. Muscle and tendon attachments, as well as periosteum and bone marrow, were removed. The remaining cortical bone was crushed with a hammer and a chisel and was then modelled under water cooling with SiC wet-grinding paper (grit 80) to obtain cuboidal test specimens (20 × 10 × 5 mm). Cylindrical test specimens (d = 6 mm2, h = 3 mm) were shaped with a Robling 800 lathe at 2000 rpm. The test specimens were stored in phosphate buffered saline (PBS) at 5 °C until use. For the shear strength testing, the cylindrical bone specimens were glued to the centre of the cuboid bone specimens (Fig. 1A). The bonded bone specimens were either tested for shear strength immediately after setting or stored at 37 °C and subjected to shear strength testing after adhesive aging of one hour, 24 h, or 7 days (*n* = 8, respectively). Shear strength testing was performed by using the test fixture (Fig. 1B) and a mechanical testing machine (Zwick/Roell Z010, Zwick GmbH & Co. KG, Ulm, Germany) at a crosshead speed of 1 mm/sec and a pre-load of 1 N. With the aid of a fixing screw in the test fixture, the bonded specimens (cylindrical and cuboid specimen bonded together) were pushed against a corresponding notch in the metal. With the help of the notch, the specimen was fixed in such a way that only the cylindrical bone body extended into the guide rail for the punch. The punch was placed on the cylindrical bone body (very close to the glue line), so that initially only the deadweight of the punch was applied to the bond. Subsequently, axial force was applied to the punch by the testing machine, causing shear stress to the bond. Measurements were taken up to the point of failure of the bond, which was registered by a drop in force. The bulk adhesives were also characterized regarding their compressive strength by preparing cuboids with a size of 6x6x12 mm, which were stored for 24 h at 37 °C prior to testing. An axial force was applied to a cuboid cement test specimen until a drop in force was registered.

The setting temperature of the adhesive cements was recorded over 30 min using a thermometer rod (Heidolph, Schwabach, Germany). The pH during setting was measured over 24 h using the InoLab Level 2 (WTW, Weilheim, Germany). For the analysis of the setting reaction and the behaviour of the organic components, the reactant powders or the already cured cements were pulverized with a mortar and placed on the ATR element of a FT-IR spectrometer (Thermo Fisher Scientific, Waltham, Massachusetts, United States). Measurements were made in a range from 4000 to 650 cm^−1^. Functional groups were identified based on the absorption bands formed by molecular vibrations and rotations as a result of infrared electromagnetic radiation. The corresponding reference spectra were taken from “Spectroscopic Methods from Organic Chemistry” by Manfred Hesse et al. [40]. Degradation experiments were undertaken by immersion of hardened adhesive samples (*n* = 3) in PBS buffer for up to 10 days while measuring their wet weight daily.

### X-ray diffraction analysis (XRD)

The adhesive cements were also analyzed for their mineral composition using a D8 Advance X-ray diffractometer (Bruker Company, Billerica, United States) with monochromatic CuKα radiation at an accelerating voltage of 40 kV in an angular range of 10–70 °. Measurements were made at a step rate of 1.2 s/step and a step size of 0.02 °. The evaluation, analysis and quantification of diffraction patterns were performed with the software products DIFFRAC.SUITE, DIFFRAC.COMMANDER, DIFFRAC.EVA and DIFFRAC.TOPAS (Bruker, Billerica, United States) using reference patterns from the International Centre of Diffraction Data database (PDF-2, 1996) for farringtonite (PDF 33-0876), magnesium oxide (PDF 43-1022 and PDF 04-0829), tetracalcium phosphate (PDF 25-1137), a-tricalcium phosphate (PDF 29-0359), and magnesium pyrophosphate (PDF 08-0038 and PDF 32-0626).

### Scanning electron microscopy (SEM) and energy dispersive X-ray analysis (EDX)

For SEM investigation, the adhesive cements were bonded to hydroxyapatite cement test specimens (made from α-TCP and 2.5% Na_2_HPO_4_) and these were then shear tested. Furthermore, the dried samples were coated with platinum at a thickness of 4.0 nm in the EM ACE600 sputter coater (Fa. Leica Camera, Wetzlar, Germany) at 10^−7^ mbar to improve SEM resolution. Scanning electron micrographs were taken on the Zeiss Crossbeam 340 scanning electron microscope (Carl Zeiss, Oberkochen, Germany) at 3.00 kV, a vacuum of 1.25 × 10^−6^ mbar, and an aperture of 30.00 µm. Microscopic images of the adhesive residues on the bone were also taken with the SteREO Discovery.V20 stereomicroscope (Carl Zeiss Microscopy GmbH, Jena, Germany) using a 81 mm PlanApo 0.63x FWD lens. The ZEN® software (Carl Zeiss Microscopy GmbH, Jena, Germany) was used for this purpose.

## Results

### Mechanical testing

**Fig. 2:**
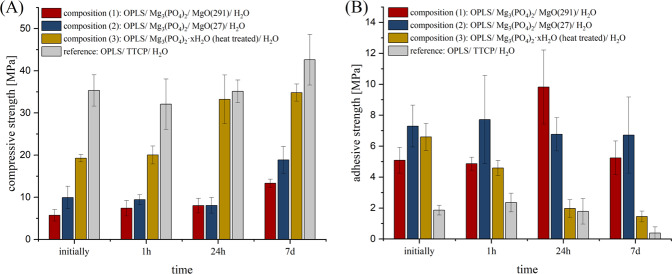
**A** Compressive strength of cement cuboids and (**B**) adhesive strength on bovine cortical bone substrates of cements made from: red: OPLS/ Mg_3_(PO_4_)_2_/ MgO(291)/ H_2_O, blue: OPLS/ Mg_3_(PO_4_)_2_/ MgO(27)/ H_2_O, yellow: OPLS/ / Mg_3_(PO_4_)_2_∙xH_2_O (heat treated.)/ H_2_O, grey: OPLS/ TTCP/ H_2_O (reference). Measurement took place initially or after 1 h, 24 h respectively 7 d hardening at 37 °C (*n* = 8, respectively)

Figure 2A shows the calculated mean values from the compressive strength tests. Composition **(1)** (OPLS/ Mg_3_(PO_4_)_2_/ MgO(291)/ H_2_O) showed a progression of compressive strength over time from 5.7 ± 1.4 MPa at initial measurement to 13.3 ± 1.0 MPa after 7 d. Composition **(2)** (OPLS/ Mg_3_(PO_4_)_2_/ MgO(27)/ H_2_O) showed a maximum compressive strength of 18.8 ± 3.1 MPa also reached after 7 d. Composition **(3)** (OPLS/ Mg_3_(PO_4_)_2_∙xH_2_O (heat treated)/ H_2_O) showed the highest compressive strength values of 34.8 ± 2.0 MPa at the last measurement time point after 7 d. Among the proprietary compositions, this one showed by far the highest compressive strength values. The reference composition **(4)** (OPLS/ TTCP/ H_2_O) showed the highest compressive strength values at the time of measurement after 7 d (42.6 ± 6.0 MPa). Figure 2B shows the calculated mean values of the bond and shear strength tests. Composition **(1)** gave the highest measured adhesive strength values within the system at the time of measurement after 24 h with 9.8 ± 2.0 MPa, as well as the highest measured values of the work as a whole. However, at the measurement time point after 7 d, lower bond strength values of 5.4 ± 1.09 MPa on average had been obtained. Initially, the system achieved an adhesive strength of 5.1 ± 0.8 MPa. Composition **(2)** gave the highest measured bond strength values at the initial measurement time point (7.3 ± 1.4 MPa). The maximum value was reached at the time point after 24 h (7.7 ± 2.9 MPa). Overall, the average readings up to the measurement after 7 d (6.7 ± 2.5 MPa) were at a similar high level. Composition **(3)** initially reached its highest adhesive strength of 6.6 ± 0.9 MPa. Temporally, a regression of the bond strength was observed. After 7 d, the system achieved an adhesive strength of 1.45 ± 0.35 MPa. The reference composition **(4)** initially achieved strength values of 1.9 ± 0.3 MPa, while after 1 h the highest measured values of 2.4 ± 0.6 MPa were obtained. After 7 d, bond strengths of only 0.4 ± 0.4 MPa were obtained, with some specimens already separating from each other during storage (evaluated as bond strength of 0 MPa). With these values, this reference composition remained clearly behind the in-house compositions in terms of bond strength.

The Pearson product-moment correlation coefficient shows a strong negative correlation between time of storage and bond strength (strong correlations defined as ≤ −0.6/ ≥0.6) for compositions **(2)**, **(3)** and the reference. In this respect, only a weak to moderate correlation of −0.25 was observed for composition **(1)**. For the correlation between the time of storage and the compressive strength, a strong positive correlation was found for all compositions investigated.

**Fig. 3:**
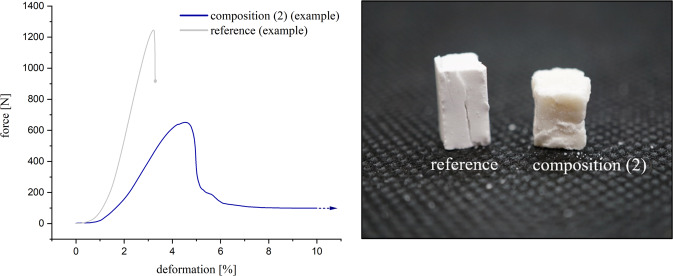
Examples of characteristic modes of failure in comparison. Blue curve/right cubus: Ductile material failure of composition (2) with plastic deformation. Grey curve/left cubus: Brittle fracture of the reference OPLS/TTCP with abrupt material failure without plastic deformation

When testing composition **(1)** or **(2)**, a cohesive failure pattern occurred permanently with adhesive residues on both parts of the joint. In the compressive strength test, the specimens of these compositions showed a ductile failure pattern. Particularly during initial testing, specimens could be massively compressed (in some cases with >50% deformation) before a relevant drop in force occurred. In contrast, brittle fractures were consistently observed in the reference group. The described deformation properties and material failures were also reflected in the stress-strain diagram (see Fig. 3). Similar to composition **(3)**, the reference also showed a predominantly adhesive fracture.

### FTIR analysis

**Fig. 4:**
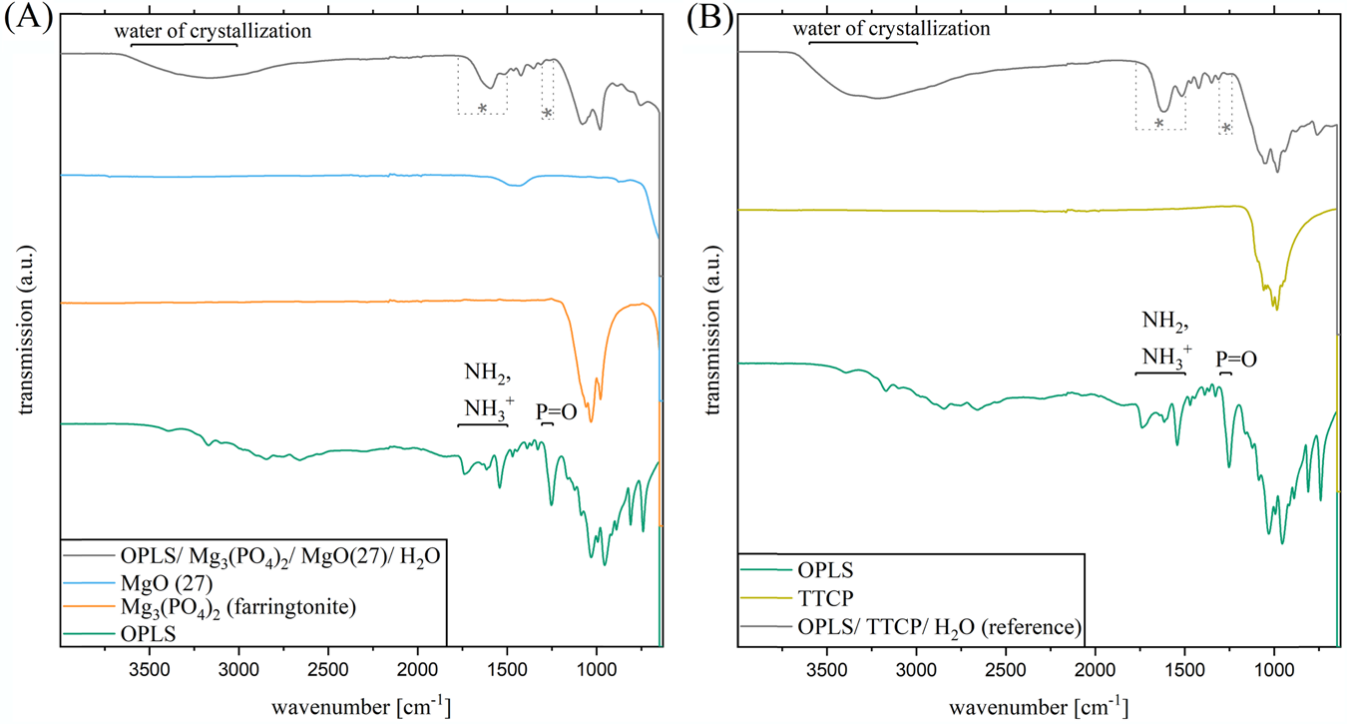
FTIR transmission spectra (incl. FTIR of the reactant powders) at the wave range of 4000 to 650 cm^−1^ of: (**A**) OPLS/ Mg_3_(PO_4_)_2_/ MgO(27)/ H_2_O. **B** OPLS/ TTCP/ H_2_O (reference)

The spectra of the measured composition **(2)** and the reference are shown in Fig. 4. Composition **(2)** is exemplary for the spectra of the remaining formulations. The deformation vibration of ─NH_3_^+^ characteristically has strong absorptions just below the 1500 cm^−1^ region and additionally between 1775 and 1600 cm^−1^. ─NH_2_ causes deformation vibrations between 1650 and 1560 cm^−1^. The corresponding peaks can be identified in the absorption spectrum of OPLS. In all cements measured, there is extinction of these peaks, suggesting a reaction of this functional group. All solid-state spectra of the set cements show a broad low-intensity band in the 3600–3000 cm^−1^ range that can be assigned to water present in the hardened adhesive. P = O bonds such as in phosphoric esters have strongly intense characteristic absorptions in the fingerprint region in the field of 1300–1250 cm^−1^. A congruent peak can be seen in the OPLS spectrum. Again, peak disappearance occurs due to setting, suggesting a reaction of the phosphoryl group. In general, it is conceivable that the functional groups of OPLS form coordinative bonds with Mg^2+^ ions during binding, resulting in chelate complexes.

### X-ray diffraction analysis

**Fig. 5:**
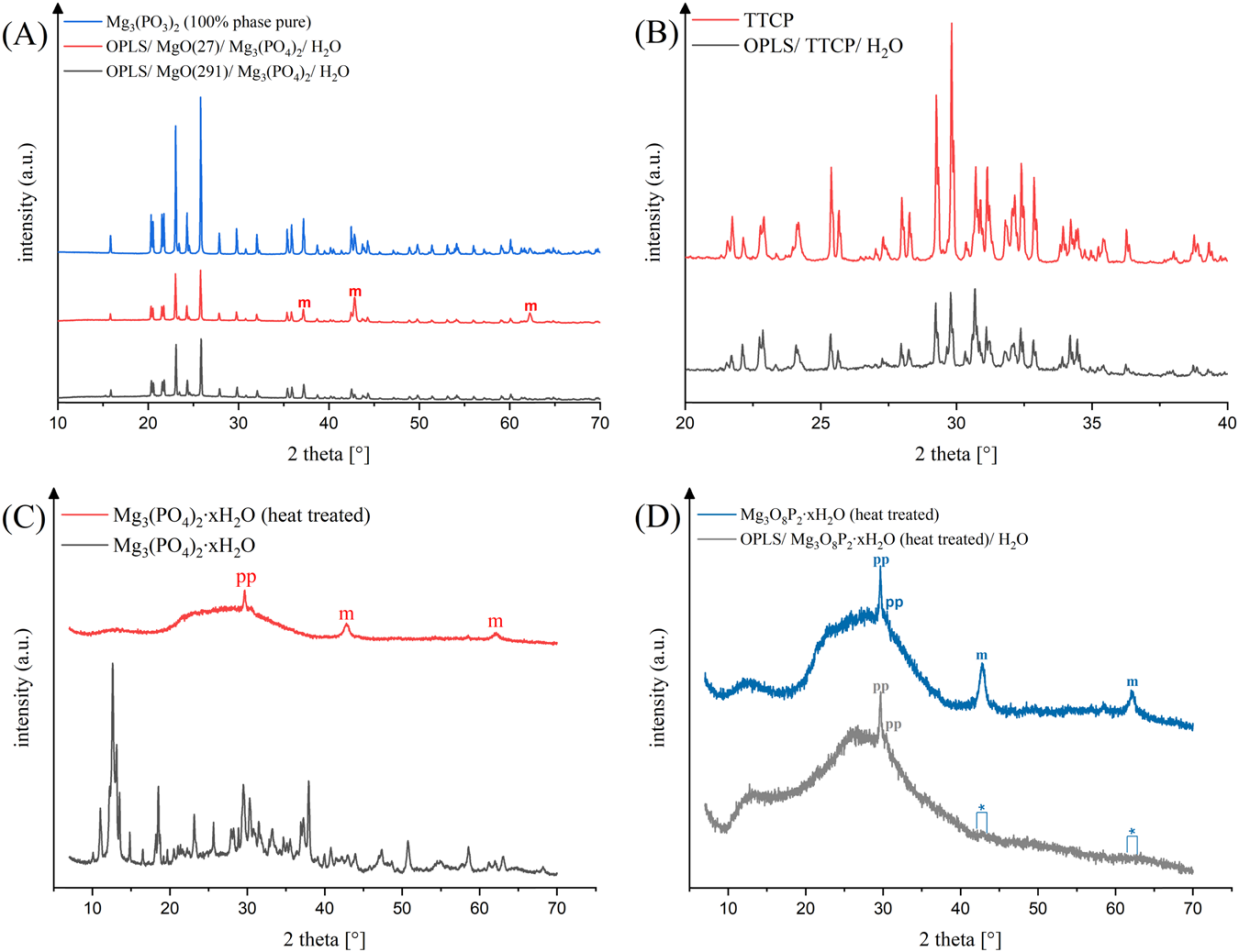
Diffraction patterns of: (**A**): OPLS/ MgO(291)/ Mg_3_(PO_4_)_2_/ H_2_O (black), OPLS/ MgO(27)/ Mg_3_(PO_4_)_2_/ H_2_O (red) and farringtonite (blue) (“m”: magnesium oxide). **B** OPLS/ TTCP/ H_2_O (grey) and TTCP (red). **C** Mg_3_(PO_4_)_2_∙xH_2_O before (grey) and after heat treatment (red) (“pp”: pyrophosphate; “m”: magnesium oxide)**. D** Mg_3_(PO_4_)_2_∙xH_2_O (heat treated) before (blue) and after (grey) reaction with OPLS and H_2_O

Figure 5 shows X-ray diffraction patterns in an angular range from 10 to 70°. In that way, diffraction patterns from the set adhesive compositions as well as from their raw powders, which have not yet reacted, are displayed. The diffractograms of compositions **(1)** and **(2)** are shown in Fig. 5A. The Mg_3_(PO_4_)_2_ powder has phase-pure farringtonite reflections. This was also demonstrated in compositions **(1)** and **(2)**, which additionally contain magnesium oxide peaks and thus are multiphase. Figure 5B depicts the phase composition of the reference and the TTCP raw powder used in the angular range of 20–40 °. The diffraction patterns correspond to α-TCP (Ca_3_(PO_4_)_2_) and TTCP (Ca_4_(PO_4_)_2_O). The same diffraction patterns result from XRD analyses of the set cement. The difference between the diffraction pattern of the TMP hydrate (Mg_3_(PO_4_)_2_∙xH_2_O) contained in composition **(3)** and the diffraction pattern of the same raw powder after heat treatment is shown in Fig. 5C. The TMP hydrate illustrates peaks for a corresponding octa and deca hydrate. Peaks for brucite (Mg(OH)_2_) and newberyite (MgHPO_4_-3H_2_O) can also be seen. Heat-treated Mg_3_(PO_4_)_2_∙xH_2_O presents itself largely amorphous with isolated peaks consistent with magnesium oxide and magnesium pyrophosphate. The peaks assigned to magnesium oxide in the heat-treated trimagnesium phosphate hydrate can no longer be found by X-ray diffraction after reaction with OPLS and water (according to composition **(3)**). (cf. Fig. 5D).

### Further material properties

**Fig. 6:**
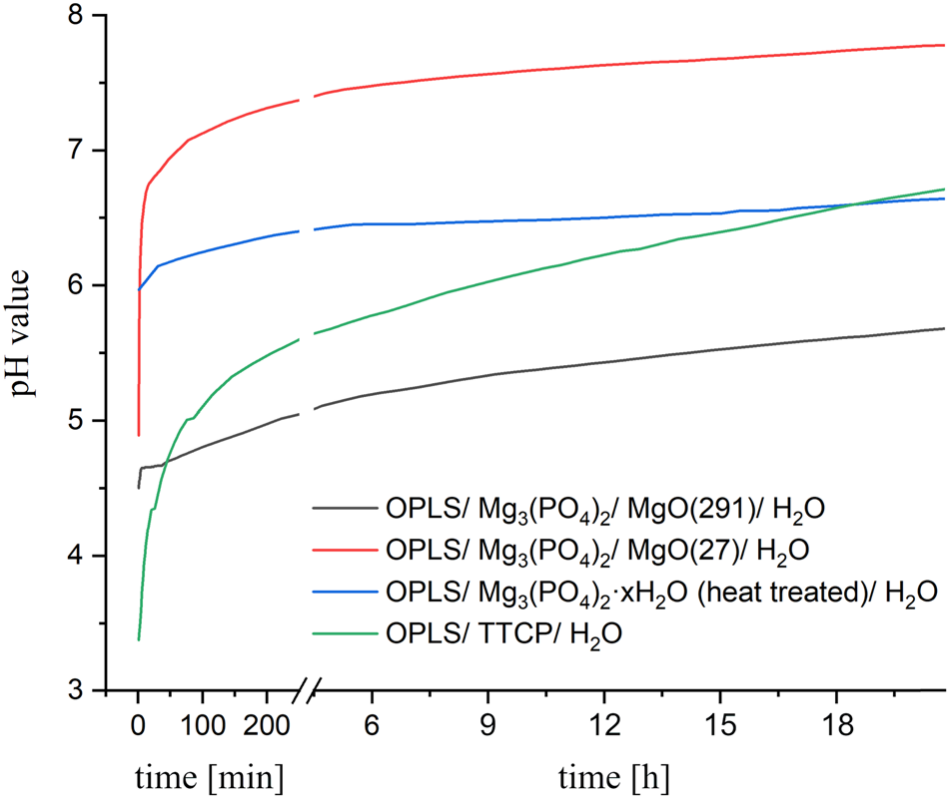
pH values over time during the setting reaction of different bone adhesives

**Fig. 7:**
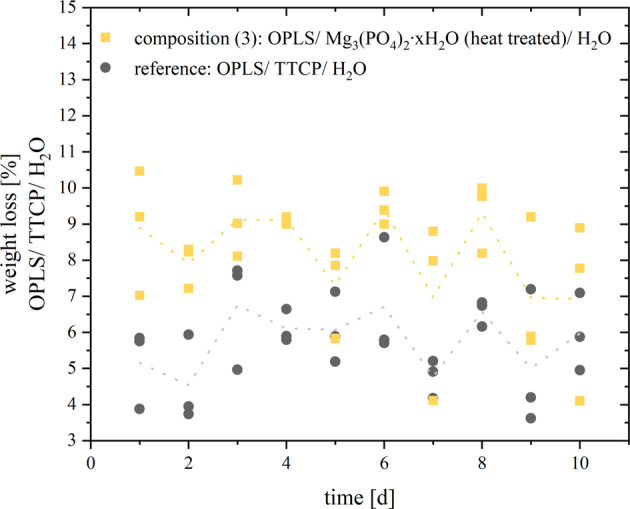
Weight loss [wt%] of composition (3) (OPLS/ Mg_3_(PO_4_)_2_∙xH_2_O (heat treated)/ H_2_O) and the reference (OPLS/ TTCP/ H_2_O) by storage in PBS over time for 10 days. The dashed line represents the course of the mean values

The maximum temperature reached during the setting reactions at room temperature was 31.4 °C for composition (**1**), 27.8 °C for composition (**2**), 30.0 °C for composition (**3**) and 32.5 °C for the OPLS/TTCP reference. The development of the pH value over time of the setting reactions is shown in Fig. 6. The greatest change in pH occurs in approximately the first 5 min of the setting reaction. After that, the values asymptotically approach a target pH. After 24 h, only minimal changes in the pH value are recorded. For the reference, this asymptotic approach is slower. Composition (**1**) and (**2**) were not dimensionally stable when stored in PBS for one week. The average weight loss during storage for 7 days was 8.19 wt.% for composition (**3**) (Fig. 7). For the reference it was 5.76 wt.%. In each case, no increase in weight loss over time was detectable. It can therefore be assumed that the weight loss takes place within the first 24 h and then stagnates.

### Scanning electron microscopy analysis

**Fig. 8:**
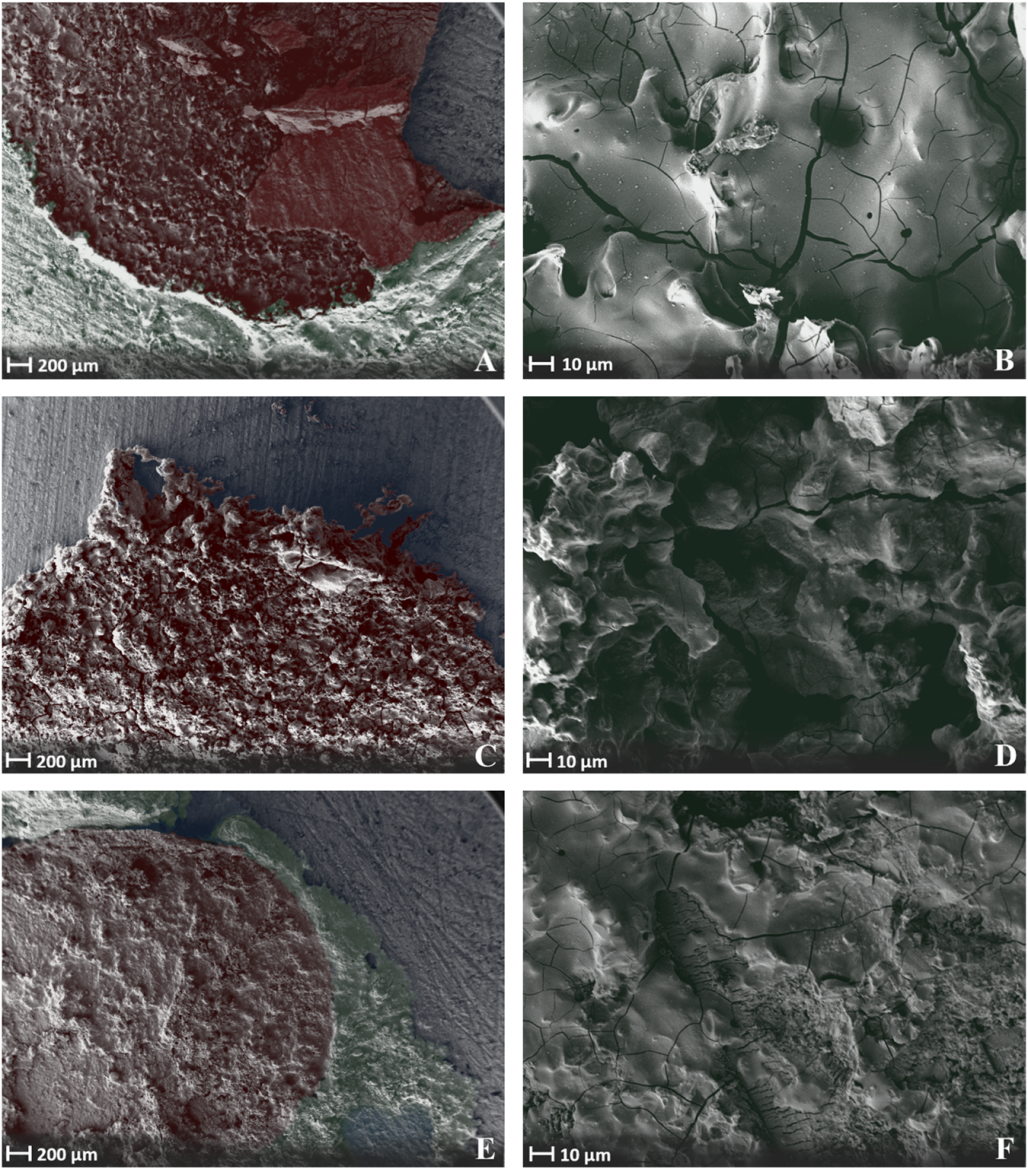
Scanning electron micrographs of an adhesive joint on hydroxyapatite specimen made of: Composition (1) (OPLS/ MgO(291)/ Mg_3_(PO_4_)_2_/ H_2_O) at 25x (**A**) and ×500 (**B**) magnification on a HA specimen after failure in the shear test. Composition (3) (OPLS/ Mg_3_O_8_P_2_∙xH_2_O(heat treated)/ H_2_O) at 25x (**C**) and ×500 (**D**) magnification. Reference (OPLS/ TTCP H_2_O) at ×25 (**E**) and ×500 (**F**) magnification. The raw images were stained by computer graphics for better orientation: Red: Sheared off adhesive joint. Green: Supernatant of the adhesive joint without mechanical testing. Blue: Hydroxyapatite test specimen machined with grit P 80

Figure 8A, B shows SEM images of adhesive joints on HA of composition **(1)** after failure in the shear test. At 25x and 500x magnification, the microscopic appearance suggests a honeycomb fracture. These cohesively fractured adhesive joints suggest a ductile fracture. At higher magnifications, crack formations of the fractured adhesive joints become visible. These could be an expression of failure during testing or of embrittlement during the necessary drying of the specimens before obtaining them in the SEM. Figure 8 shows images of composition **(3)** (C-F) and the reference composition (**4**) (E-F), which are similar in their microscopic morphology. The structure of these mostly adhesively fractured surfaces (81,3% for composition **(3)** and 100% for the reference adhesive failure mode) would be consistent with a more brittle fracture pattern.

## Discussion

**Fig. 9:**
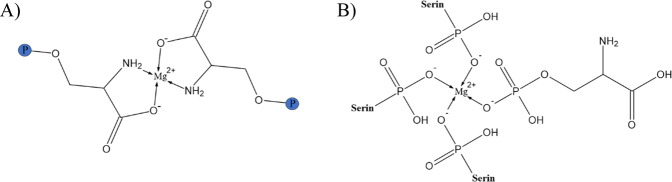
Conceivable configurations for coordinative bonds of phosphoserine with Mg^2+^: **A** Coordinative bonds between Mg2+ and amino or carboxyl groups. **B** Coordinative bonds between Mg^2+^ and phosphoryl groups

The focus of the work was the development of adhesive organoceramic cements based on OPLS and magnesium phosphates or oxides. It was found that the combination of OPLS and crystalline farringtonite was not sufficiently reactive to obtain self-setting adhesives. This was solved by adding MgO as a highly reactive Mg^2+^ source similar to the use as a setting accelerator in other MPC systems based on coordinative bonds via phosphate groups [18]. Two different types of MgO with varying reactivity were used (composition **(1)** and **(2)**), which required relatively large amounts of OPLS to achieve the desired adhesive properties. A further adhesive (composition **(3)**) was based on an amorphous magnesium phosphate, which was obtained by a temperature treatment of commercially available TMP hydrate (which is composed of phases such as Mg_3_(PO_4_)_2_·10H_2_O, Mg_3_(PO_4_)_2_·8H_2_O (bobierrite), Mg(OH)_2_ (brucite), MgHPO_4_·3H_2_O (newberyite) according to XRD analysis). TMP hydrate converts to a primarily amorphous powder with sufficient reactivity, whereas residual MgO (obtained from thermal decomposition of brucite) disappeared after reaction with OPLS, supporting the described affinity of OPLS for MgO. As reference, we used a mixture of TTCP and phosphoserine as it is known from literature (composition **(4)**) [39]. All compositions were mixed with water to initiate the setting process. A slight temperature increase to 27.8–32.5 °C was observed, which is far below the temperature range at which heat necrosis and permanent damage of tissue can occur, as known to happen for exothermically setting PMMA bone cements [41–43]. In addition, the pH during setting was found to be in the range of 6–7.5. These pH values are comparatively close to the physiological pH of the body, where cells and their metabolic processes function best. Hence side-effects caused by an acidic pH such as inhibited osteoblastic mineralization [44] or the release of proinflammatory cytokines [45, 46] are not to be expected. The setting mechanism of the adhesives is based on a reaction of the functional carboxyl, amino and phosphoryl groups of the phosphoserine (see FT-IR spectra in Fig. 4) with Mg^2+^ to form chelate complexes. Conceivable configurations for such coordinative bonds of phosphoserine are shown in Fig. 9. Furthermore, coordinative bonds to the Ca^2+^ of the inorganic bone mineral are likely the decisive factor for the adhesive properties of the system. Kesseli et al. [47] also suggested coordinative bonds in calcium phosphate / OPLS systems and the formation of a calcium-phosphoserine monohydrate phase as the predominant parameter for the setting of such adhesives. In contrast, X-ray diffraction analyses within our study did not detect any newly formed crystalline magnesium phosphate phases in the sense of a conventional cementation reaction (Fig. 5A, B), which supports the presumed setting mechanism.

Like the TTCP/OPLS reference, adhesives based on magnesium phosphate minerals demonstrated a high adhesion on cortical bone substrates over an ageing period of 7 d. The initial shear strength for composition **(1)**–**(3)** ranges from 5.1 ± 1.1 MPa to 7.3 ± 1.4 MPa with practically no change in adhesion for the MgO containing compositions **(1)** and **(2)** after 7 d. In contrast, composition **(3)** based on a thermally treated TMP powder showed a continuous decrease in adhesion over time, while the TTCP/OPLS reference had an adhesive strength of ~ 2 MPa during the first 24 h, which decreased to <1 MPa after 7 days. The latter is in agreement with works from Kirillova et al. [22] who determined an adhesive strength of ~ 2 MPa to cortical bone for a similar TTCP/OPLS composition (referring to Tetranite ®). It is significant to note, that the mechanical performance of the adhesives under compressive load followed the opposite trend. Here, the TTCP/OPLS reference as well as the TMP based composition **(3)** showed compressive strengths of 20–40 MPa, which was 2–4 times higher than that of compositions **(1)** and **(2)**. This inverse relationship between the inherent strength of the adhesives and their adhesion to the bone substrates might be supported by the more ductile character of the latter compositions, which in turn resulted in a predominant cohesive failure mode. This finding is supported by a study from Methew et al. [48] who investigated the adhesive properties of phosphoserine-bearing calcium phosphate cements and yielded the highest adhesive strengths at relatively high phosphoserine contents of 23–72 mol% (corresponding to 15–46 wt%) [48]. In our study, those compositions with the highest phosphoserine content (compositions **(1)** and **(2)**) exhibit a consistently cohesive fracture failure, with comparatively low compressive strength at the same time. A ductile character of the adhesive seems to be advantageous especially for the application in a biomechanical system with a dynamic mechanical loading situation. The reference, on the other hand, is clearly brittle (Fig. 3 and Fig. 8E, F) and the fracture behaviour is predominantly adhesive. Overall, the novel mineral adhesives from our study clearly exhibit nearly fourfold better adhesive properties than the currently known bone adhesives. The bond strengths are also significantly higher than the 0.2 MPa defined by Weber and Chapman [8] as the minimum bond strength of a clinically applicable bone adhesive. To the author’s knowledge, there is currently no commercially available bone adhesive for use on humans. Therefore, only commercial tissue adhesives tested on bone can be used for comparison. The author himself has previously tested various tissue adhesives using the test method presented here [49]: For adhesives such as BioGlue® (two-component adhesive made from bovine serum albumin and glutaraldehyde), a novel MPC (containing farringtonite, MgO and phytic acid), or a light-curing adhesive (consisting of star-shaped NCO-sP(EO-*stat*-PO), PEGDMA, newberyite and camphorquinone), bond strengths above 1.5 MPa were never achieved (from initial measurements to measurements after 7 days of storage in PBS solution). For the tissue adhesives TruGlue® and Histoacryl®, which belong to the cyanoacrylates, the highest measured values under the same conditions were slightly below 4 MPa. As mentioned above Tetranite® [19] and OsStic™ [20] are designated bone glues intended for commercialization and have been submitted to other author-specific testing procedures. They are based on the reaction between OPLS and α-TCP (OsStic™) or TTCP (Tetranite®). For the combination of OPLS and α-TCP, adhesive strengths of up to 2.5–4 MPa were recorded when cured in an aqueous environment [21]. The reaction between TTCP and OPLS resulted in an adhesive strength of ~2 MPa to cortical bone substrates [22].

There is currently no test standard for bone adhesives. Test methods vary between different research studies [50], as improvised and laboratory- or company-specific test methods become necessary. The advantages of the test method used here over other testing processes have been discussed in detail in a previous work of the author [49]. Overlapping bonding according to DIN EN 1465 for conventional glues, for example, can hardly be realized for the “material bone”. The test specimen geometries used, on the other hand, can be realized from cortical bovine femur. The test procedure offers clear geometries and a force vector nearly parallel to the adhesive joint.

Accounting for the above-mentioned aspects, the bond strengths of the cementitious compositions presented here appear remarkably high in comparison. One reason for the improved adhesive properties compared to the other designated bone adhesives could be the pH value during setting. In biological systems, phosphorylated amino acids complex metal ions only with their phosphate group at low pH. At neutral pH, the coordination site changes and the amino and carboxyl groups form complexes with metal ions [51–55]. Even at high pH, the phosphate group then does not form complexes. However, Mg^2+^, which prefers coordinative bonds to oxygen, can complex with these negatively charged phosphate groups [54–56]. Transferred to the chelate formation in the investigated biomineral adhesive cements, it is significant that these cements, which comprise Mg^2+^ as metal ion set in the course of a rather neutral pH value (especially composition **(2)** and **(3)**), which is slightly higher than in the comparative formulations with TTCP (see Fig. 7). It is conceivable that, analogous to the biological situation, carboxyl and amino groups predominantly complex at this higher pH. Due to the high oxygen affinity, it would be conceivable that Mg^2+^ ions would nevertheless complex with the phosphate groups, which would result in more functional groups (amino and carboxylate) being available in the system than in chelate complexes with Ca^2+^ (reference composition), which would favour the adhesive properties.

In biocomplexes, non-covalent bonds within coordinative systems are also very important [57]. Due to the acid-base equilibrium, phosphate groups as ligands donate protons and non-covalent complexes can form [38]. Positive centre of such interaction could be the amino group of phosphoserine itself [58]. Jastrzab et al. [38] observed the strongest non-covalent bonds in a system of phosphoserine and biogenic amines pH-dependent at a value close to 7.0, at which the phosphate group, as well as the carboxyl group, were deprotonated, whereas the amino group was still present in a protonated state. Composition **(2)** and composition **(3)** in particular set neutrally near a pH of 7 (see Fig. 7).

Although not tested in this study, we would assume that the OPLS-MgP adhesives will at least have cytocompatible properties as all components are non- cytotoxic and various combinations of magnesium phosphate minerals [59–63] as well as OPLS-calcium phosphate adhesives [22, 64] have been tested thoroughly in vitro and in vivo. Since magnesium phosphate cements showed superior biodegradability [65] compared to calcium phosphate, active resorption by osteoclasts is considered to be possible. Regarding the described properties, the investigated cement compounds of phosphoserine and magnesium phosphates or oxides thus appear to be promising bone adhesives.

The cement compositions presented here are easily scalable. OPLS, TMP hydrate or magnesium oxide are commercially available and comparatively inexpensive chemicals. Heat treatment as well as the production of farringtonite are rather easy processes and can be carried out in large quantities.

## Conclusion

This work demonstrated that a combination of magnesium phosphate minerals with OPLS was successful to achieve self-setting formulations with a high adhesiveness to cortical bone. The reaction kinetics were adjusted by either adding caustic calcined reactive magnesium oxides or by calcination of amorphous trimagnesium phosphate hydrate. An inverse relation between the compressive strength of the bulk adhesive and the adhesion to cortical bone was found, combined with higher ductility and a predominant cohesive failure mode. The novel adhesives not only outperformed previous materials based on TTCP/OPLS mixtures directly after fixing cortical bone substrates, but also maintained their adhesion over an ageing course of 7 days. FTIR and XRD analyses suggested the formation of chelate complexes between the functional groups of phosphoserine and Mg^2+^ as the setting mechanism. Coordinative bonds to Ca^2+^ from bone mineral as well as electrostatic interactions are presumably co-responsible for the high adhesive strength. Apparently, the pH-value plays a decisive role since it is important for the coordination sites of phosphorylated amino acids. It is conceivable that the described bonding and reaction mechanisms may also work with other phosphorylated biomolecules such as phosphothreonine or phosphotyrosine as already known for calcium phosphate cement modification, which may open the way for further improvements of the adhesives. Due to their unique mechanical performance and handling properties, as well as excellent setting and curing times, the presented biomineral adhesive cements may create new perspectives in clinical treatments of comminuted fractures. Future research efforts on bone adhesives made of phosphoserine and Mg^2+^ seem to be very promising. The use in comminuted fractures or the use in non-weight-bearing defects are possible fields of application of such bone adhesives.
